# Automated Aerospray Hematology PRO staining shows good agreement for mature leukocytes but limited diagnostic reliability for immature forms: comparison with manual May-Grünwald-Giemsa staining

**DOI:** 10.11613/BM.2026.010708

**Published:** 2026-02-15

**Authors:** Ana Nikler, Matea Tomas, Andrea Saračević, Vanja Radišić Biljak

**Affiliations:** 1 University Hospital Sveti Duh, Department of Medical Laboratory Diagnostics, Zagreb, Croatia; 2 University of Zagreb, Faculty of Kinesiology, Department of Sport and Exercise medicine, Zagreb, Croatia

**Keywords:** Aerospray Hematology PRO Slide Stainer, verification, May Grünwald Giemsa, manual technique

## Abstract

**Introduction:**

Microscopic examination of peripheral blood smears remains essential step in hematology diagnostics, requiring reliable and standardized staining techniques. This study evaluated performance of Aerospray Hematology PRO Slide Stainer (ELITechGroup Inc., Utah, USA) in comparison with manual May-Grünwald-Giemsa (MGG) technique.

**Materials and methods:**

Forty K_2_EDTA-whole-blood smears were prepared in duplicate and stained using both methods. Hundred samples flagged by Siemens Advia 2120i for atypical lymphocytes (ATYPS), immature granulocytes (IG), blasts and nucleated red blood cells were analyzed for diagnostic accuracy. Precision was assessed using three K_2_EDTA-whole-blood samples, where 12 smears *per* sample were evaluated. Manual differential counts of 100 white blood cells (WBC) *per* slide were performed by experienced laboratory scientist. Data distribution was assessed using Kolmogorov-Smirnov test. Method comparison was performed using Bland-Altman and Passing-Bablok analyses, while sensitivity and specificity were calculated for ATYPS, IG and blasts.

**Results:**

Precision results met acceptable criteria for all WBC subpopulations. No significant differences were observed for mature WBCs: intercept - 4.0 (- 13.8 to 3.0) slope 1.0 (0.9 to 1.2) for neutrophils; intercept - 1.5 (- 9.3 to 1.9), slope 1.1 (0.9 to 1.3) for lymphocytes; intercept 1.0 (- 2.0 to 1.6), slope 1.0 (0.9 to 1.4) for monocytes; intercept 0.0 (- 1.5 to 1.3), slope 1.0 (0.5 to 1.7) for eosinophils. Staining of mature WBCs was comparable, showing no significant differences in nuclear or cytoplasmic morphology. While immature WBCs, particularly myelocytes, displayed fewer granules and lighter nuclear staining with Aerospray. Diagnostic accuracy was unsatisfactory for classifying ATYPS (Se = 73%, Sp = 60%), IG (Se = 63%, Sp = 50%) and blasts (Se = 63%, Sp = 100%), whereas erythrocyte and platelet morphology were unaffected.

**Conclusions:**

Aerospray Hematology PRO is suitable for mature WBC populations. However, manual MGG staining remains necessary for reliable evaluation of immature and pathological cells.

## Graphical abstract

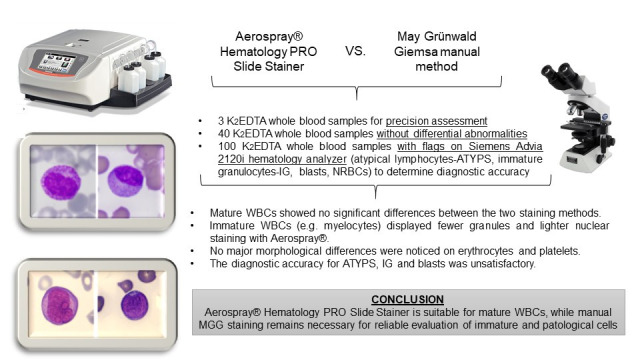

## Highlights

• Aerospray provided comparable staining to May-Grünwald-Giemsa (MGG) for mature white blood cells (WBCs)

• Manual MGG staining remains necessary for reliable evaluation of immature cells

• Diagnostic accuracy was unsatisfactory for atypical lymphocytes, immature granulocytes and blasts

• Immature WBCs had reduced granularity and lighter nuclear staining with Aerospray

• Erythrocyte and platelet morphology were unaffected by the staining method

## Introduction

Microscopic examination of peripheral blood smears remains an essential step in laboratory hematology diagnostics despite the overgrowing laboratory automatization (*1*). Careful morphological assessment of blood cells provides critical information for the detection of hematological abnormalities, recognition of abnormal cell forms, and identification of immature or pathological cell populations, which may be crucial for establishing a diagnosis and monitoring disease progression or treatment response. Therefore, high-quality and standardized blood smear preparation and staining are indispensable for ensuring accurate microscopic interpretation and minimizing inter-laboratory variability.

Conventional manual preparation and staining of peripheral blood smears, most commonly performed using the May-Grünwald-Giemsa (MGG) technique, remains widely used in routine laboratory practice (*2*). However, manual smear preparation and staining are time-consuming procedures, highly dependent on the experience of laboratory professionals and susceptible to inter- and intra-operator variability. To address these limitations, automated smear stainers have been introduced to improve workflow efficiency, reduce human error, and ensure better reproducibility of staining quality. A wide range of commercially available automated smear stainers are integrated with hematology analyzers, facilitating more rapid and standardized preparation of peripheral blood smears. Numerous studies have investigated the performance of such integrated systems and their impact on morphological assessment (*3*-*9*).

The aim of this study was to compare the staining quality and diagnostic suitability of peripheral blood smears prepared using the Aerospray Hematology PRO Slide Stainer (ELITechGroup Inc., Utah, USA) and the standard manual laboratory procedure based on MGG technique. The evaluation was performed using routine complete blood count (CBC) samples, with a focus on cellular morphology visualization and the reliability of leukocyte differential analysis.

## Materials and methods

### Study design

This verification study was performed in a clinical hematology laboratory following routine diagnostic workflow. The study was conducted in October 2023 at the Department of Medical Laboratory Diagnostics, University Hospital Sveti Duh (Zagreb, Croatia). It comprised assessment of staining precision, method comparison, and evaluation of diagnostic performance for selected leukocyte populations. The Institutional Ethics Committee approved the use of residual patient blood samples for the purposes of laboratory method evaluation and quality improvement (approval number 01-2013).

### Sample selection

A total of 120 K_2_EDTA samples (Becton Dickinson, Franklin Lakes, USA, 2mL) were included in the study, out of which 40 of them previously analyzed on a Siemens Advia 2120i automated hematology analyzer (Siemens, Marburg, Germany), without differential abnormality, were selected for staining comparison. Additionally, 20 samples *per* each of the following flags were included in the study of diagnostic accuracy: atypical lymphocytes (ATYPS), immature granulocytes (IG), blasts and nucleated red blood cells (NRBCs) (N = 80). Also, three samples with different leukocyte differential counts (neutrophils > 70%, lymphocytes > 50%, monocytes > 12%) were selected for precision determination. Blood samples were obtained from both inpatient and outpatient populations, encompassing patients from various hospital departments, in order to reflect routine clinical hematology practice.

### Blood smear preparation

Two blood smears from each of the selected samples were simultaneously prepared by inverting the sample tube 8-10 times before pipetting 25 microliters for each blood smear. The blood sample was dispensed on the slide approximately 5-7 millimetres from the frosted part of the slide. A spreading slide with frosted edges was lightly pressed on the slide at a 30° to 45° angle then moved backwards to contact the blood drop so that it spread along the width of the slide edge, and was moved forward in a motion so that insures the smear is not too thick not too thin. The smear was covering two thirds of the glass with a well rounded, bullet like edge. After drying at room temperature for 25-30 minutes the slides were stained both by MGG manual technique and by Aerospray Hematology PRO Slide Stainer.

### Manual dip staining May-Grünwald Giemsa method

Manual MGG staining was performed using a dip-staining procedure according to the manufacturer’s instructions (Sigma-Aldrich), consisting of four sequential steps (*10*):

1. Slides are immersed in May-Grünwald eosin methylene blue solution by Merck (Darmstadt, Germany) for 5 minutes;
2. Smears are rinsed in pH 6.8 buffer for approximately 1-1.5 minutes;
3. Following, slides are placed in 5% buffered Giemsa azur eosin methylene blue solution by Merck for 20 minutes;
4. Final step includes rinsing in demineralised water and left to air dry before microscoping.

### Staining by Aerospray Hematology PRO Slide Stainer

Aerospray staining instrument utilizes a controlled six-step staining process. The instrument has four operating possibilities: Rapid, Wright-Giemsa; May Grunwald Giemsa and Custom. Extensive modifications of the staining programs are possible in order to improve individual staining steps (*11*). After the initial setup of the Aerospray HematologyPRO Slide Stainer device, all solutions were prepared according to the manufacturer’s instructions. Aerospray uses four solutions, out of which two are ready to use, while two are prepared manually (ELITechGroup Inc, Logan, USA). The blood slides are sprayed directly by each solution thus reducing the risk of cross-contamination. Firstly, the slides were sprayed with the fixator, the intensity setting we chose for the fixation of the slides was level 3, afterwards the slides were sprayed simultaneously with Thyazine (ELITechGroup Inc, Logan, USA) and Eosin (ELITechGroup Inc, Logan, USA) in a concentrated staining cycle, the ratio of red/blue was 65/35. After a mid-rinse, the diluted staining cycle started. The diluted cycles ratio of red/blue was 60/40 with the dilution of stain/ buffer ratio of 30/70, dilute intensity was at a level 2, and end rinse intensity was at a level 6 to try to prevent staining the reverse side of the slides. The staining cycle comes to an end with a drying cycle. The slides were fully stained and ready for microscopic examination in less than 10 minutes after loading into the dying carousel. All of the used materials and ways of preparation are listed in Table 1.

**Table 1:** Comparison of staines used for morphology staining between automated Aerospray Hematology PRO Slide Stainer and standard manual May Grünwald Giemsa technique

	**Staines**	**Preparation**
May-Grünwald Giemsa (MGG) manual technique	May-Grünwald’s eosin-methylene blue solution	Ready to use
	Giemsa azur eosin methylene blue solution	10 mL of concentrated Giemsa solution is diluted in 190 mL of the pH 6.8 buffer
	Buffer tablets pH 6.8 for preparing buffer solution	The buffer pill is dissolved in 1 liter of demineralised water
Aerospray Hematology PRO Slide Stainer	Aerospray Hematology Pro Reagent A, Buffer (pH 7.2)	3 mL of the Hematology Pro Reagent A, Buffer with 500 mL of distilled water
	Hematology Reagent B: Thiazin Stain	Ready to use
	Aerospray Hematology Pro Reagent C, Eosin	Ready to use
	Hematology Reagent D - Aerofix Fixative with methanol	Mix of 500 mL of methanol and 15 mL of a concentrate Aerofix

For descriptive purposes, selected workflow-related characteristics of manual MGG staining and the Aerospray Hematology PRO Slide Stainer are presented in Table 2. These characteristics include approximate slide preparation time, hands-on time, level of process standardization, and routine maintenance requirements.

**Table 2:** Comparison of workflow-related characteristics of manual May-Grünwald-Giemsa staining and Aerospray Hematology PRO Slide Stainer

**Parameter**	**Manual May-Grünwald-Giemsa staining**	**Aerospray Hematology PRO Slide Stainer**
Staining principle	Manual dip staining	Automated spray-based staining
Total slide preparation time*	Approximately 40-45 minutes	Approximately 5-7 minutes
Hands-on time	High (continuous manual handling)	Low (automated process)
Throughput	Limited	High
Process standardization	Operator-dependent	High (automated reagent application)
Protocol flexibility	Limited manual adjustment of staining steps	Extensive programmable staining options
Routine maintenance	Every-day reagent preparations and cleaning	Daily and weekly maintenance according to manufacturer’s recommendations
*Approximate times recorded under routine diagnostic workflow; not derived from a formal time-motion study.		

### Verification procedure

#### Precision

To avoid possible imprecision errors, prior the comparison studies a short precision study was performed. For three peripheral K_2_EDTA-whole blood samples, 12 smears were prepared from each sample. A complete 12-position carousel of the Aerospray Hematology PRO Slide was loaded and all smears were stained simultaneously under identical conditions. Each slide was subsequently examined microscopically, and the standard deviation (SD) and coefficient of variation (CV) were calculated for the main leukocyte (WBC) subpopulations: neutrophils, lymphocytes, monocytes, and eosinophils. Acceptability criteria were established according to biological variability defined by EFLM Biological Variation Database (*12*).

#### Method comparison

All 120 paired stained slides were independently evaluated by a single experienced medical laboratory scientist, blinded to the staining method, in order to minimize potential inter-observer variability. For each sample, a manual differential count of 100 leukocytes was performed on both manually stained MGG slides and Aerospray-stained slides, thereby simulating the usual routine laboratory practice. Digital microscopy using the CellaVision DC-60 system was used for illustrative documentation of selected representative blood smears in order to support visual comparison of morphological features. CellaVision was not available for routine use at the time of the study and was therefore not used for statistical evaluation.

#### Diagnostic accuracy

Diagnostic accuracy of Aerospray staining for ATYPS, IG and blasts was determined by comparison with manual microscopic evaluation of MGG-stained peripheral blood smears, which served as the reference (gold standard) method. Diagnostic performance was expressed as sensitivity and specificity. Acceptability criteria were defined according to Vis *et al.*, with sensitivity > 80% and specificity > 95% for ATYPS, sensitivity > 90% and specificity > 70% for IG, and sensitivity and specificity > 95% for blasts (*13*).

### Statistical analysis

The comparison of WBC differential counts in samples without morphological abnormalities was performed by using Bland-Altman and Passing Bablok statistical analyses. Prior to method comparison, data distribution normality was assessed using the Kolmogorov-Smirnov test. While the diagnostic accuracy in classifying ATYPS, IG and blasts was performed by a 2x2 diagnostic table out of which sensitivity and specificity were calculated. Statistical analysis was performed in MedCalc Statistical Software version 22.014 (MedCalc Software Ltd, Ostend, Belgium).

## Results

The precision study results were satisfactory, with all WBC subpopulations meeting predefined acceptable criteria (Table 3). The RBCs stained by Aerospray were a rich red color and no effect on distinction of any RBC morphological abnormalities were observed.

**Table 3:** Results of the precision study of WBC subpopulations in Aerospray-stained peripheral blood smears

	**Neutrophils**			**Lymphocytes**			**Monocytes**			**Eosinophils**		
	**S1**	**S2**	**S3**	**S1**	**S2**	**S3**	**S1**	**S2**	**S3**	**S1**	**S2**	**S3**
Mean (%)	75.3	37.3	66.7	10.6	53.2	16.5	7.0	6.4	15.5	1.9	3.4	1.3
SD	3.5	4.3	2.1	2.8	4.0	2.0	2.0	2.1	1.3	0.8	1.2	0.5
CV (%)	4.7	11.6	3.2	26.9	7.5	12.3	27.9	32.2	8.5	41.4	36.3	37.2
Acceptance criteria for CV* (%)	32.5			28.0			33.2			41.4		
*acceptance criteria defined by EFLM Biological Variation Database (12). S1 - sample 1. S2 - sample 2. S3 - sample 3. SD - standard deviation. CV - coefficient of variation.												

**Figure 1:**
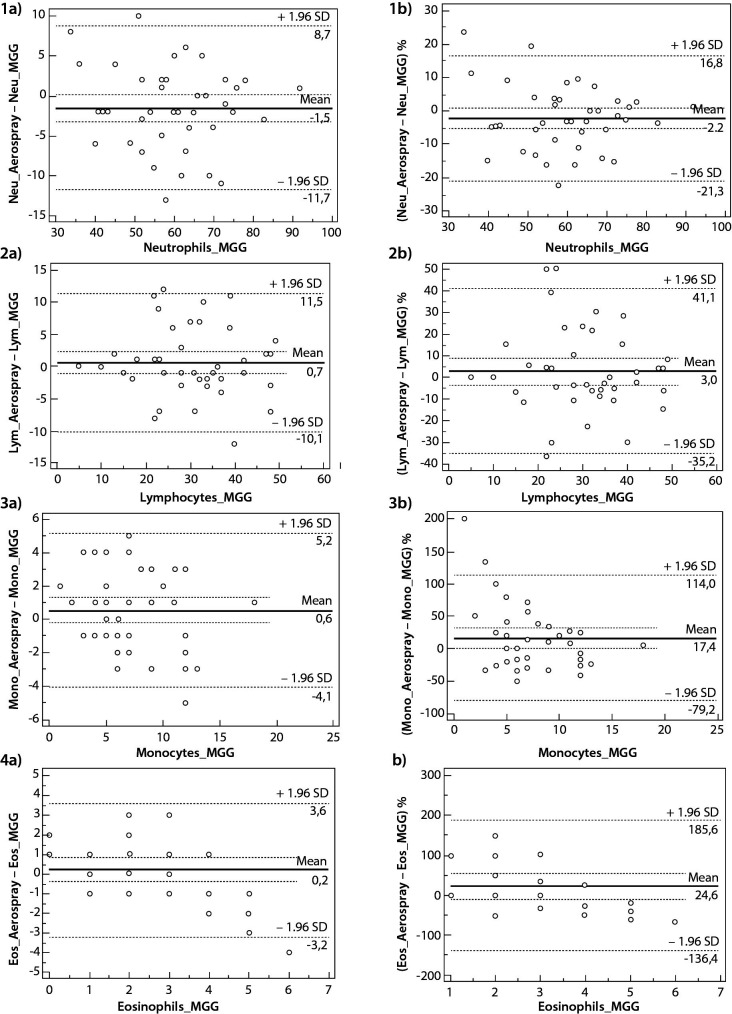
Bland-Altman plots comparing wgite blood cell differential counts obtained from manually stained May Grünwald Giemsa (MGG) and Aerospray-stained peripheral blood smears. Panels A and B represent absolute differences and relative bias, respectively, for neutrophils (*1*), lymphocytes (*2*), monocytes (*3*), and eosinophils (*4*).

**Figure 2:**
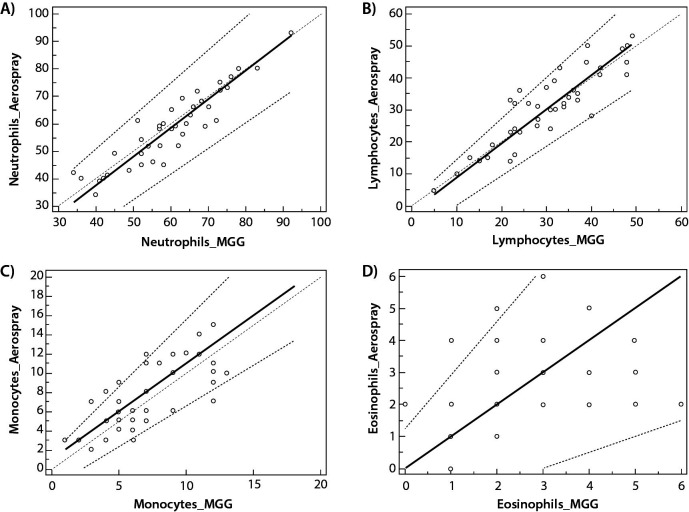
Passing Bablok scatter plots of white blood cell differential counts obtained from manually stained May Grünwald Giemsa (MGG) and Aerospray-stained peripheral blood smears. A) neutrophils, B) lymphocytes, C) monocytes, D) eosinophils.

No statistically significant differences were observed between paired slides for WBC subpopulations, as determined by Bland Altman and Passing Bablok statistical analyses, with normal data distribution confirmed by the Kolmogorov Smirnov test (Figures 1-2). Data for basophils were not sufficient for Passing Bablok analysis due to low cell counts (2% as highest value). An unsatisfactory diagnostic accuracy was observed in classifying ATYPS (Se = 73%, Sp = 60%, SOTA > 80% / > 95%), immature granulocytes (Se = 63%, Sp = 50%, SOTA > 90% / > 70%) and blasts (Se = 63%, Sp = 100%, SOTA > 95% / > 95%) as presented in Table 4. Samples flagged for NRBCs by the hematology analyzer did not show erythroblasts on microscopic examination of peripheral blood smears and were therefore excluded from further analysis.

**Table 4:** Diagnostic accuracy of Aerospray staining for atypical lymphocytes, blasts and immature granulocytes, compared with the May-Grünwald-Giemsa method

	**ATYPS (%)**	**Acceptable** criteria* (%)	**BLAST (%)**	**Acceptable** criteria* (%)	**IG (%)**	**Acceptable** criteria* (%)
Sensitivity (Se) (95% CI)	73 (45-92)	> 80	63 (25-91)	> 95	63 (35-85)	> 90
Specificity (Sp) (95% CI)	60 (15-94)	> 95	100 (74-100)	> 95	50 (7-93)	> 70
*defined by Vis *et al* as State-of-the-art (SOTA) criteria (13). ATYPS - atypical lymphocytes. BLAST - blast cells. IG - immature granulocytes. CI - confidence interval.						

There was no significant difference in mature WBCs as far as the color differential of the nuclei and cytoplasm. However, there was a slight difference in the coloring of the toxic granules found in some of the smear duplicates. Aerospray staining of toxic granules was more red than purple and less represented than in the MGG stained slides.

**Figure 3:**
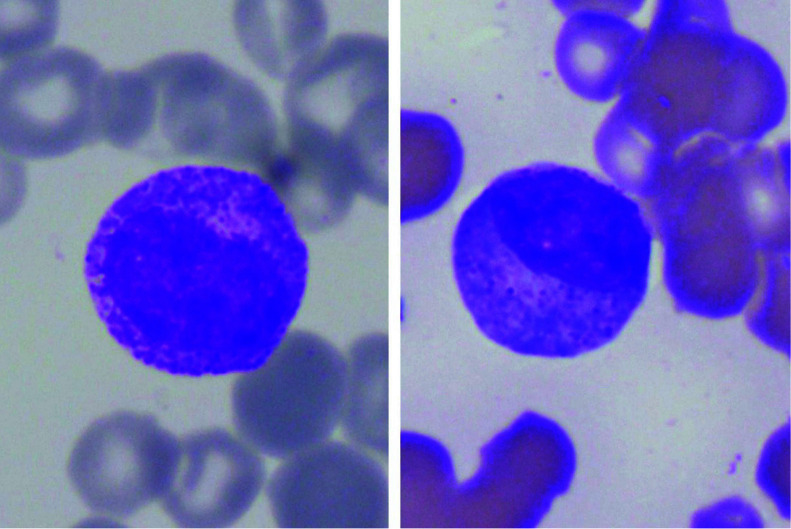
The comparison of myelocyte morphology in a peripheral blood smear stained by standard manual May Grünwald Giemsa technique (left) and by Aerospray Hematology PRO Slide Stainer (right). Both depicted cells were taken from the same sample.

**Figure 4:**
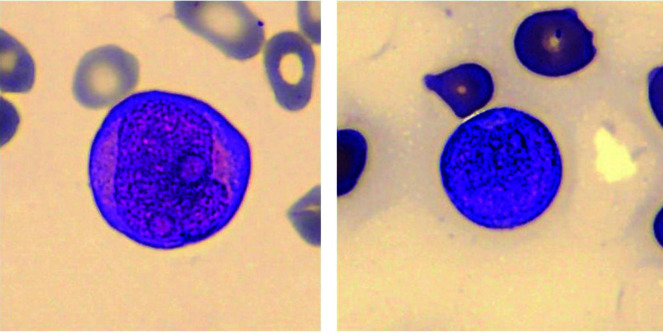
The comparison of blast morphology in a peripheral blood smear stained by standard manual May Grünwald Giemsa technique (left) and by Aerospray Hematology PRO Slide Stainer (right). Both depicted cells were taken from the same sample.

Immature WBCs were noticeably different. Although the cytoplasm was standardly stained, there were significantly fewer primary granules represented in the slides stained by Aerospray compared to the manual MGG technique. The MGG stained the immature WBCs with a nucleus a bit more intensely with a dark purple, but the primary granules were by comparison far more noticeable in color and in number unlike in the firstly mentioned method that especially goes as far as myelocytes differential (Figure 3). The differences in depiction of primary granules differed vastly between the two slides. There was no difference with staining atypical lymphocytes, the nucleus was a purple/light pink colour in both methods, with overwhelming clear cytoplasm, noticeable red granules were present and a darkened blue edge. There was slight difference with staining of the blasts. The MGG staining resulted in a darker purple colouring of the nucleus and a noticeable nucleolus discolouring, whereas Aerospray staining of the nucleus was lighter in color which made it slightly harder to notice the nucleoli, typically found in blasts (Figure 4). Cytoplasm was clear, a light blue color with a darkened blue edge on both staining methods.

Although the main focus of the comparison study was on the WBC’s and RBC’s morphology, no major morphological differences were noticed on the platelets.

## Discussion

An earlier publication of the Aerospray staining system in hematology was published by Duchayne and Aldebert in 2008 (*14*). In that study, the authors demonstrated that, after appropriate adjustment of staining parameters, Aerospray staining could produce peripheral blood and bone marrow smears with overall morphological appearance comparable to the conventional MGG method. However, their evaluation was primarily focused on qualitative assessment of staining characteristics and did not address diagnostic performance or the reliability of leukocyte differential assessment in routine clinical samples. In contrast, the present study provides a contemporary, clinically oriented re-evaluation of Aerospray Hematology PRO Slide Stainer using routine peripheral CBC samples selected based on analyzer flags. Unlike previous report, our study integrates statistical comparison, diagnostic accuracy analysis, and focused assessment of immature and pathological leukocyte populations according to current state-of-the-art performance criteria.

Our results demonstrate that, in samples without morphological abnormalities, Aerospray-stained slides showed good agreement with manually stained MGG slides for mature leukocyte populations. However, in samples flagged for potential morphological abnormalities, the automated staining method demonstrated limited diagnostic accuracy for the identification and differentiation of immature and pathological leukocyte forms. This distinction, which was not addressed in earlier studies, is particularly relevant in modern hematology laboratories where morphological review is increasingly triggered by analyzer flags and where accurate identification of immature cells is critical for clinical decision-making.

The main practical advantage of the Aerospray Hematology PRO Slide Stainer is undisputably its speed. While the standard manual MGG technique requires a minimum of 40-45 minutes to produce a quality stained slide, with Aerospray Hematology PRO Slide Stainer turnover time can be reduced to 5-7 minutes. Additionally, the uniform spreading of the dye enables better standardization of the whole process. Coupled with the automated microscopy analyzer, such as CellaVision, the analysis and classification of the stained slides also becomes standardized and consistent which leads to a clean, efficient, and smooth workflow with excellent results, as shown by Verdoes *et al*. (*15*). In their study, various Aerospray staining settings were assessed in combination with automated WBC classification using the CellaVision DM 1200. The authors predefined that a staining setting would be considered acceptable if the automated WBC classification achieved ≥ 95% accuracy as verified by an experienced laboratory technician. However, it is important to note that manual staining techniques were not included in their evaluation, nor were the smears reviewed manually under the microscope.

Beyond preparation time, the two staining methods differ in several workflow-related aspects that are relevant for routine laboratory practice. The main advantages of the Aerospray Hematology PRO Slide Stainer include a high degree of process standardization, reduced hands-on time, and suitability for high-throughput laboratories. Automated reagent application minimizes operator-dependent variability and facilitates integration into routine diagnostic workflows.

According to International Council for Standardization in Haematology (ICSH) guidelines, substantial differences may exist between digital microscopy systems that utilize manually prepared and stained blood smears and those employing automated slide makers and stainers (*16*). Although automated systems generally provide standardized smear preparation and staining, comparative studies evaluating differences in morphological details and color consistency between these two approaches remain limited. Previous reports have noted that blood smears prepared automatically may display cells that appear larger and thinner, with potential chromatic variations compared to cells stained using panoptical manual techniques (*7*, *17*, *18*). In the presence of abnormal cells or in pediatric samples rich in lymphocytes, such variations in cell size, thickness, and coloration may contribute to misclassification, often leading to an overestimation of blast cells (*19*). Our study observed significant differences in the staining of immature WBCs, particularly myelocytes, where Aerospray staining showed fewer and less distinct primary granules and lighter nuclear coloration compared to the manual MGG method. Furthermore, the diagnostic accuracy for atypical lymphocytes and blasts was unsatisfactory when using Aerospray, while the morphology of mature WBCs was comparable between the two methods, with no significant differences in nuclear or cytoplasmic staining. To enhance laboratory interpretation, the ICSH guidelines recommend that manufacturers provide high-quality reference images comparing manually and automatically prepared smears, along with clear explanations of expected morphological and chromatic differences (*16*).

Although a full digital morphology comparison was not performed, the observed staining-related differences in immature leukocyte populations are clinically relevant in the context of modern hematology workflows, where digital morphology systems are increasingly used as a decision-support tool (*20*, *21*). Suboptimal visualization of primary granules and lighter nuclear staining may adversely affect both manual and digital classification, particularly in samples flagged for immature or pathological cells.

This study has several limitations. Digital morphology was not available for routine use at the time of the study and therefore could not be applied systematically or included in diagnostic performance analysis. Slide evaluation was performed by a single experienced laboratory scientist, reflecting routine laboratory practice but precluding assessment of inter-observer variability. Manual differential counts were limited to 100 WBCs *per* slide in order to simulate routine workflow and did not follow CLSI H20-A2 recommendations for evaluation of leukocyte differential counting methods (*22*). Additionally, NRBCs were not evaluated because samples flagged for nucleated red blood cells did not demonstrate erythroblasts upon microscopic review. Pediatric and neonatal samples were not analyzed separately, and no further optimization of staining parameters was attempted, which may limit the applicability of the results to other populations or staining conditions. Cost *per* sample was not systematically assessed as such analysis is highly dependent on local pricing, reagent contracts, workload, and laboratory organization. The coloring of the RBCs in manually stained slides with MGG technique was slightly grayish, than prefereably light pink, perhaps due to not so optimal buffer pH, which was not checked during the verification process. However, that did not influence the distinction of any RBC morphological abnormalities, as stated before.

Neverthless, under the applied staining protocol, Aerospray Hematology PRO Slide Stainer demonstrated comparable performance to manual MGG staining for samples with mature leukocyte populations. However, the automated staining method showed insufficient diagnostic accuracy for the reliable identification and classification of immature and pathological leukocyte forms. Consequently, manual MGG staining remains indispensable when evaluating samples in which immature or pathological cells are expected.

In conclusion, these findings define the practical scope and limitations of the automated staining method as applied in this study.

## Data Availability

All data generated and analyzed in the presented study are included in this published article.
